# AI-assisted design synthesis and human creativity in engineering education

**DOI:** 10.3389/frai.2026.1714523

**Published:** 2026-01-20

**Authors:** Mariza Tsakalerou, Saltanat Akhmadi, Aruzhan Balgynbayeva, Yerdaulet Kumisbek

**Affiliations:** Department of Civil and Environmental Engineering, Nazarbayev University, Astana, Kazakhstan

**Keywords:** artificial intelligence, creativity, design synthesis, engineering education, human–AI collaboration

## Abstract

The growing integration of AI into educational and professional settings raises urgent questions about how human creativity evolves when intelligent systems guide, constrain, or accelerate the design process. Generative AI offers structured suggestions and rapid access to ideas, but its role in adopting genuine innovation remains contested. This paper investigates the dynamics of human-AI collaboration in challenge-based design experiments, applying established creativity metrics: fluency, flexibility, originality, and elaboration in order to evaluate outcomes and implications in an engineering education context. Through an exploratory quasi-experimental study, a comparison of AI-assisted and human-only teams was conducted across four dimensions of creative performance: quantity, variety, uniqueness, and quality of design solutions. Findings point to a layered outcome: although AI accelerated idea generation, it also encouraged premature convergence, narrowed exploration, and compromised functional refinement. Human-only teams engaged in more iterative experimentation and produced designs of higher functional quality and greater ideational diversity. Participants’ self-perceptions of creativity remained stable across both conditions, highlighting the risk of cognitive offloading, where reliance on AI may reduce genuine creative engagement while masking deficits through inflated confidence. Importantly, cognitive offloading is not directly measured in this study; rather, it is introduced here as a theoretically grounded interpretive explanation that helps contextualize the observed disconnect between performance outcomes and self-perceived creativity. These results bring opportunities and risks. On the one hand, AI can support ideation and broaden access to concepts; on the other, overreliance risks weakening iterative learning and the development of durable creative capacities. The ethical implications are significant, raising questions about accountability and educational integrity when outcomes emerge from human-AI co-creation. The study argues for process-aware and ethically grounded frameworks that balance augmentation with human agency, supporting exploration without eroding the foundations of creative problem-solving. The study consolidates empirical findings with conceptual analysis, advancing the discussion on when and how AI should guide the creative process and providing insights for the broader debate on the future of human–AI collaboration.

## 1 Introduction

### 1.1 Generation, synthesis and creativity

The emergence of AI-driven code generation and synthesis technologies has revolutionized how creative problem-solving is approached in software development and engineering design. These systems demonstrate unprecedented capabilities in combining existing knowledge patterns to produce novel code solutions, design concepts, and technical implementations. However, as AI tools become increasingly sophisticated at both creating new content and incorporating disparate information sources, fundamental questions arise about their impact on human creative processes.

Modern AI systems excel at synthesis, which the Cambridge Dictionary defines as “the mixing of different ideas, influences, or things to make a whole that is different, or new.” This capability allows them to systematically recombine existing knowledge from vast repositories, mirroring how advanced code-generation tools integrate patterns, functions, and algorithmic structures to create novel implementations (Haase and Hanel, 2023; Kirkpatrick, 2023). However, the distinction between this sophisticated recombination and genuine creativity, defined as “the ability to produce or use original and unusual ideas,” remains contested.

While AI systems demonstrate remarkable capabilities in combining knowledge in increasingly sophisticated ways, their capacity for true innovation is fundamentally constrained by their reliance on pre-existing data (Habib et al., 2024). This limitation becomes particularly significant in engineering education, where students must master both the effective use of existing solutions and the development of genuinely innovative approaches to novel problems. Some scholars argue that even AI’s ability to integrate remote and unrelated knowledge domains enables innovative outcomes that produce “original and unused ideas” (Lee and Chung, 2024), suggesting that cross-domain integration capabilities have important implications for both code generation and broader engineering design processes.

Foundational work by Guilford (1950) and Torrance (1966) emphasizes that creative performance depends on divergent thinking processes, specifically the fluency, flexibility, and originality of ideas generated. These classic insights examine whether AI-assisted teams engage in the expansive, exploratory ideation required for genuine creative development. Understanding how these AI capabilities affect human creative development requires careful examination, particularly in educational contexts where the goal extends beyond producing outputs to developing students’ own creative problem-solving abilities.

### 1.2 Human and AI creativity

The tension between AI capabilities and human creativity has led to extensive empirical research comparing these abilities using established measures such as the Alternative Uses Task (AUT) and Torrance tests (Góes et al., 2023; Stevenson et al., 2022; Summers-Stay et al., 2023). Moreover, empirical findings have prompted researchers to develop new terminology describing different forms of AI-involved creativity, reflecting the evolving relationship between human designers and intelligent systems: artificial creativity (Ivcevic and Grandinetti, 2024; Runco, 2023), computational creativity (or creative computation) (Colton and Wiggins, 2012), and assisted creativity or co-creativity (Wingström et al., 2024).

The concept of assisted creation processes is particularly relevant to code generation and design synthesis. Recent research suggests we are entering “a new era of ‘assisted creativity,’ where AI serves not as an independent creator but as a collaborative creative agent” (Habib et al., 2024; Vinchon et al., 2023). This collaborative model reflects how modern programming increasingly involves human-AI partnerships, where developers guide intelligent tools to generate code meeting specific requirements while adding their own insights to solve complex problems. At the same time, prior work in engineering education has shown that while AI tools are perceived by both students and faculty as valuable for explanation, content generation, and learning support, they also raise concerns related to academic integrity, overreliance, and diminished critical engagement (Lukhmanov et al., 2025).

Recent philosophical work further clarifies the limits of AI-assisted creativity by foregrounding the ontological boundaries of machine generation. Lockhart (2025) argues that while AI systems can simulate novelty through recombination, creativity remains fundamentally human because it is grounded in embodied experience, emotional depth, and moral agency. From this perspective, AI-generated outputs lack the intentionality, self-authorship, and existential risk-taking that characterize human creative acts. Lockhart’s framework complements empirical models of assisted and co-creativity (Ivcevic and Grandinetti, 2024; Wingström et al., 2024) by emphasizing that augmentation does not imply equivalence. In educational contexts, this distinction is particularly important: when AI tools guide ideation without reflective engagement, learners may produce technically novel outputs while disengaging from the cognitive and ethical processes that sustain authentic creative development. This human-centered perspective provides a conceptual lens for interpreting empirical patterns such as premature convergence and cognitive offloading observed in AI-assisted design teams, reinforcing the need for pedagogical frameworks that preserve human agency alongside technological support.

These collaborative frameworks have significant implications for engineering education, where students must learn to work effectively with intelligent systems while developing their own problem-solving capabilities. Following Haase and Hanel (2023), while philosophical debates about the nature of innovation continue, the more pressing educational question concerns how widespread AI use affects human creative development in practical design and programming contexts.

### 1.3 Creativity metrics and measurement approaches

Given the evolving nature of human-AI collaboration in creative tasks, establishing reliable assessment metrics becomes crucial for understanding educational impacts. Design and engineering contexts have developed sophisticated approaches applicable to both traditional and AI-assisted creative processes.

The most widely accepted framework evaluates innovation across two primary dimensions: novelty and usefulness (Amabile, 1982; Doshi and Hauser, 2024; Harvey and Berry, 2022). This dual-dimension approach proves particularly relevant for AI-assisted contexts, as it separates idea generation from practical application, paralleling the difference between AI’s generative capabilities and human evaluative judgment. Novelty assesses how much an idea departs from expectations, while usefulness reflects practicality and relevance, including appropriateness, feasibility, and implementability (Doshi and Hauser, 2024). These dimensions translate effectively into code-generation contexts, where novel algorithmic approaches must meet functional requirements and coding standards.

Building upon this foundation, researchers have also adapted classical creativity metrics originally developed by Torrance (1966) for assessing human creative thinking:

- fluency (quantity of ideas),
- flexibility (diversity or variety of categories of ideas),
- originality (uncommonness or uniqueness of ideas), and
- elaboration (development depth or quality of ideas).

As shown in Table 1, among the various dimensions employed in recent empirical studies, four metrics appear most frequently across different task types and provide comprehensive coverage of creative output assessment.

**Table 1:** Overview of recent empirical studies on the human and AI creativity.

Study	Fluency	Flexibility	Elaboration	Originality	Additional dimensions	Task type	Aim and sample
Habib et al. (2024)	+	+	+	+		AUT^a^	Impact of AI on undergraduate students (*n* = 56)
Guzik et al. (2023)	+	+		+		TTCT^b^	Creativity of AI vs. undergraduate students (*n* = 24)
Hubert et al. (2024)	+		+	+		AUT, DAT^c^	Creativity of AI vs. undergraduate students (*n* = 151)
Haase and Hanel (2023)	+			+		AUT	Creativity of AI vs. undergraduate students (*n* = 100)
Koivisto and Grassini (2023)				+		AUT	Creativity of AI vs. undergraduate students (*n* = 256)
Lee and Chung (2024)				+	Appropriateness	AUT	Impact of AI on creativity (*n* = 233)
Doshi and Hauser (2024)					Novelty, Usefulness	Writing	Impact of AI on writers (*n* = 293)

a AUT: alternative uses task.

b TTCT: Torrance tests of creative thinking.

c DAT: divergent association task.

Notably, recent empirical studies do not limit the application of these metrics to human creativity assessment alone but expand their scope to evaluate AI-generated outputs, human-AI collaborative processes, and comparative analyses between traditional and AI-assisted creative work (Table 1). Studies examining human and AI creativity demonstrate considerable variation in which dimensions are measured and how they are operationalized across different task types and populations (Guzik et al., 2023; Habib et al., 2024; Marrone et al., 2024). This heterogeneity in measurement approaches reflects both the evolving nature of the field and ongoing debates about appropriate assessment frameworks for AI-augmented creative work.

However, AI-augmented contexts introduce new complexities. As Wingström et al. (2024) note, creative AI “contests the issues regarding novelty, autonomy, and authorship, as AI’s creativity is often evaluated via the outcome it produces.” These concerns become particularly acute in educational settings, where goals extend beyond producing outputs to developing students’ own creative capabilities.

### 1.4 AI integration in engineering education: opportunities and challenges

The integration of AI tools in engineering education reflects broader global trends, with countries such as Singapore, Estonia, Australia, New Zealand, and Scotland leading implementation efforts (Gabriel et al., 2022; Marrone et al., 2022). These initiatives recognize that preparing students for AI-augmented professional environments requires hands-on experience during education. However, this transformation raises critical questions about pedagogical approaches, particularly regarding prompt engineering skills and problem-definition capabilities, which remain essential in both code-generation and design contexts (Haase and Hanel, 2023).

Despite extensive research on AI creativity and growing educational interest, a significant gap remains in understanding how AI usage impacts human creativity in collaborative educational settings. Most existing studies focus on philosophical questions about whether AI can be creative, but fewer examine the practical question of how AI assistance affects human creative development in team-based contexts. This gap is particularly pronounced in engineering education research, where design synthesis and creative problem-solving represent fundamental skills students must master.

As Haase and Hanel (2023) note, “Continued research and development of GAI [Generative AI] in creative tasks is crucial to fully understand this technology’s potential benefits and drawbacks in shaping the future of creativity.” The limited research on team-based AI-assisted creativity leaves important questions unanswered: How do AI tools affect collaborative creative processes that characterize real-world engineering practice? Do AI-assisted teams produce more creative solutions, or does AI assistance reduce individual creative development? Moreover, the parallel between design creativity and programming creativity suggests that findings from design-focused research may inform broader questions about AI’s impact on creative problem-solving across technical domains.

To address these critical gaps, this exploratory study investigates two interconnected research questions:

RQ1 examines how AI assistance affects team creativity across four established dimensions and identifies which aspects of creativity are most influenced by AI usage. Specifically:

- RQ1a: Do AI-assisted teams differ from control teams in creative output across the four dimensions?
- RQ1b: Which dimension of creativity shows the most significant difference between AI-assisted and non-AI-assisted conditions?

RQ2 examines educational implications by analyzing participants’ self-perceptions of creative capability, exploring how AI assistance affects individual confidence in creative problem-solving and its implications for educational practice. Specifically:

- RQ2a: Does AI assistance lead students to overestimate their creative performance compared to objective creativity metrics?
- RQ2b: How does this perception–performance discrepancy differ between AI-assisted and non-AI teams?

This study addresses the research gaps through a systematic comparative investigation contrasting AI-assisted teams with human-driven teams in collaborative engineering design tasks. Our approach builds on established creativity assessment frameworks, specifically adapted for educational contexts, to evaluate team performance across four dimensions that capture both divergent thinking aspects (Quantity, Variety) and convergent thinking aspects (Quality), with Uniqueness bridging generative and evaluative processes. Through this comparative evaluation, the research explores the cognitive, functional, and ethical implications of AI-assisted design, contributing to understanding not only how AI affects team creativity but also broader questions about AI-driven synthesis and automated problem-solving—the issues central to the evolving relationship between human creativity and artificial intelligence in technical domains.

## 2 Materials and methods

### 2.1 Study design and experimental conditions

This exploratory, comparative, quasi-experimental investigation was embedded within a 90-min workshop focused on creative engineering practices (Photographic documentation of the workshop set-up and environment is presented in Appendix A). The study employed a between-subjects design to examine how AI assistance influences team creativity in collaborative engineering design tasks, utilizing the Catapult Challenge adapted from Seelig’s “creativity-under-constraint” framework (Seelig, 2017).

A convenience cohort of volunteers, comprising postgraduate students, early-career lecturers, and industry mentors, self-organized into eight teams of two to three members each. Demographically, participants were predominantly engineering-focused (85% reported an Engineering background), 62% male and 38% female, with 30% under 34 years and 70% aged 45 or older, representing both emerging and established professionals. To ensure unbiased allocation, the session chair prepared 12 shuffled, opaque envelopes (seven designated as “AI group” and five designated as “non-AI group”), and immediately before the task, one representative from each self-organized team drew an envelope to determine random condition assignment. This allocation method was blind to participants’ background, experience, and prior exposure to design tasks, providing baseline equivalence across these variables. All participants had no prior exposure to the experimental task and were clearly informed of the AI usage restrictions applicable to their assigned condition.

The experimental task required teams to design and construct a functional tabletop catapult capable of launching a marble at least 50 centimeters within a 45-min timeframe. Each team received an identical construction kit containing wooden craft sticks, rubber bands, plastic spoons, masking tape, a marble, scissors, and a metric measuring tape. Teams assigned to the AI-assisted condition received uninterrupted browser access to ChatGPT-4 (web version 2025-03-13) along with a standardized prompt sheet that read: “Suggest three distinct catapult concepts using only craft sticks, rubber bands, and a spoon. For each concept, list the mechanical principle, a simple ASCII sketch, and one improvement tip.” These teams retained complete autonomy to refine, modify, or disregard ChatGPT’s suggestions according to their judgment. Control teams were permitted to sketch freely and consult non-LLM web resources such as Wikipedia or YouTube, but received explicit reminders that large language models were prohibited. No financial incentives were provided to ensure participation remained voluntary and intrinsically motivated.

### 2.2 Measurement framework and experimental procedure

The study employed both individual-level and team-level assessments to capture different dimensions of creative performance within a structured experimental protocol. The experimental session began with pre-survey completion (approximately 10 min), during which individual participants provided baseline measures of creative self-efficacy using a four-item, three- to five-point Likert scale, alongside single-item measures of divergent-thinking confidence, problem-solving efficacy, and innovative-thinking self-ratings. These instruments were adapted from the 4C Creativity Assessment and administered via Google Forms. In the scope of this study, Little-c or everyday creativity was addressed, aiming to assess “original and appropriate ideas or products in the context of everyday life and interactions” (Ivcevic and Grandinetti, 2024).

Following a five-minute briefing that included specific AI-usage guidelines for applicable teams, participants engaged in the 45-min Catapult Challenge with unrestricted access to a designated testing station for prototype evaluation. Upon task completion, all participants immediately completed the post-survey (approximately 10 min), which included team-level creativity assessments using the four-dimensional framework synthesized from the metrics established in the literature review:

1. Quantity (fluency)—“How many catapult designs did your team consider?” (5-point Likert scale ranging from 1 design to 5+ designs).
2. Variety (flexibility)—“Did designs employ different mechanical principles?” (3-point Likert scale: 0 = no variety, 1 = medium variety, 2 = distinct variety).
3. Uniqueness (originality/novelty)—“How unique was your final design?” (3-point Likert scale: 0 = standard design, 1 = somewhat unique design, 2 = unique design).
4. Quality (usefulness)—“How well did your catapult work?” (3-point Likert scale: 0 = failed, 1 = worked somewhat, 2 = worked well).

Two independent raters transferred categorical responses to a master coding sheet, achieving strong inter-coder agreement (*κ* = 0.94). Both surveys are presented in Appendix B.

The session concluded with a brief, ungraded showcase and debrief discussion, though no feedback from this final stage was incorporated into the dataset to maintain the integrity of the collected measures.

### 2.3 Data analysis

All analyses were conducted using R 4.3 software. Data were exported to CSV format, screened for missing values (less than 2% across all items), and ordinal-coded for statistical analysis. Descriptive statistics, including means with standard deviations and frequency percentages, summarized individual and team outcomes. Given the small cell counts inherent to the experimental design, Mann–Whitney U tests were used to assess differences in post-survey creativity scores between AI-assisted and control teams. Pre-to-post changes within each cohort were evaluated using Wilcoxon signed-rank tests. Statistical significance was established at *α* = 0.05 using two-tailed tests, with no correction for multiple comparisons given the exploratory nature of the investigation.

## 3 Results

All analyses were conducted at two complementary levels: team-level design outcomes (Quantity, Variety, Uniqueness, and Quality) and individual-level creative self-ratings recorded before and after the Catapult Challenge. The sample size is AI = 7 teams; non-AI = 5 teams.

### 3.1 Team-level design outcomes

Mann–Whitney U tests revealed no statistically significant differences between AI-assisted and non-AI teams at *α* = 0.05. However, effect sizes ranged from small to large (*r* = 0.26–0.56), with Uniqueness approaching significance and the remaining three showing a trend. The consistent direction of effects suggests non-AI teams outperformed AI-assisted teams across all measures, though statistical power was limited by the small sample size. (Mann–Whitney U tests did not reveal statistically significant differences between conditions; this outcome should be interpreted in light of the very small number of teams).

**Figure 1:**
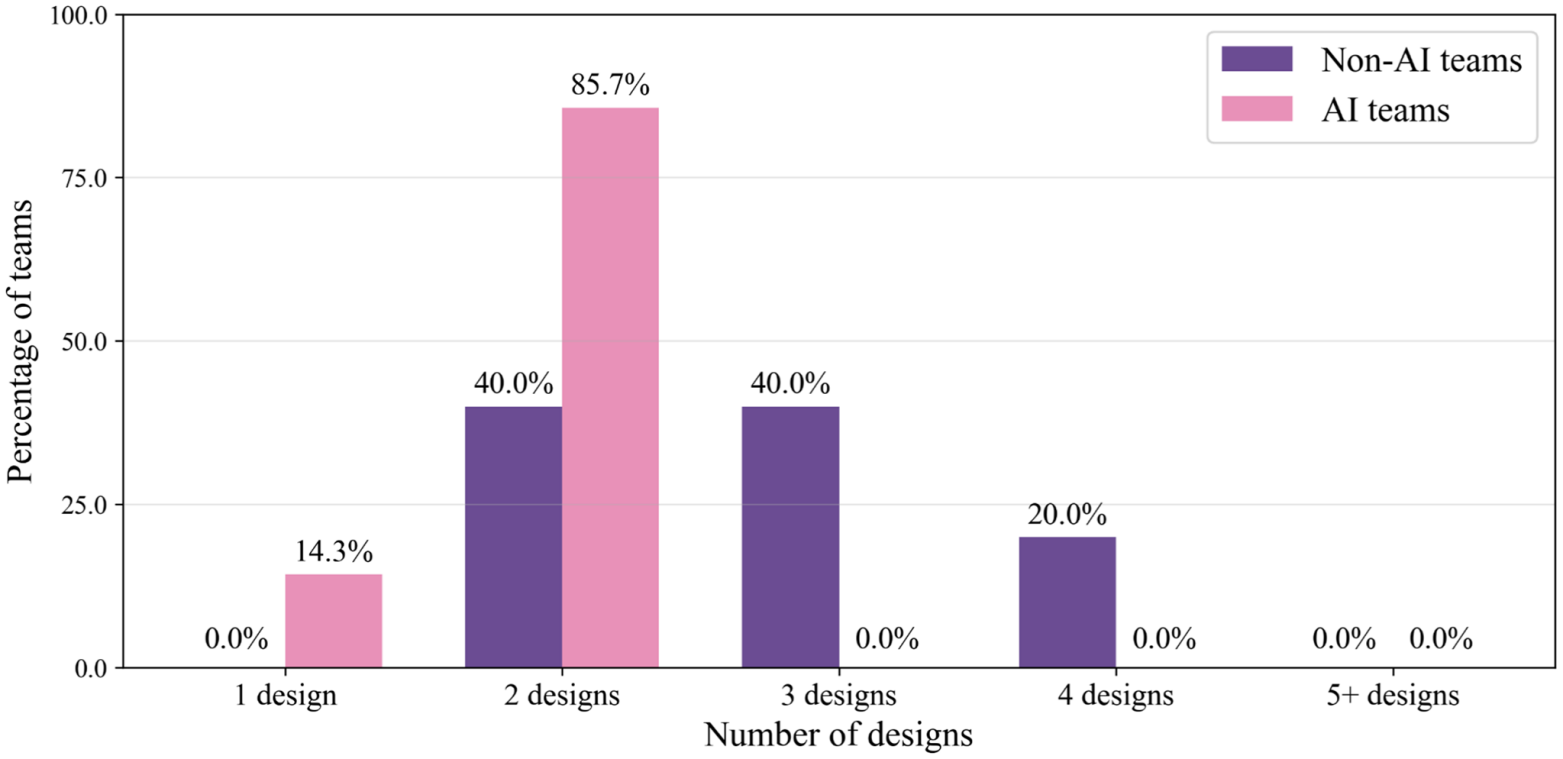
Number of catapult designs considered by AI-assisted and non-AI control teams.

A further descriptive-comparative analysis also revealed consistent patterns favoring non-AI teams across multiple creativity dimensions. As illustrated in Figure 1, design exploration, or the Quantity, differed markedly between conditions. Non-AI teams demonstrated greater design breadth, with 60% (3 out of 5) considering three or more design variants compared to 0% of AI-assisted teams (*U* = 6 with *p* = 0.073 and effect size *r* = −0.52). This pattern suggests, but does not clearly demonstrate, that AI assistance may have encouraged premature convergence on initial concepts rather than supporting divergent exploration.

**Figure 2:**
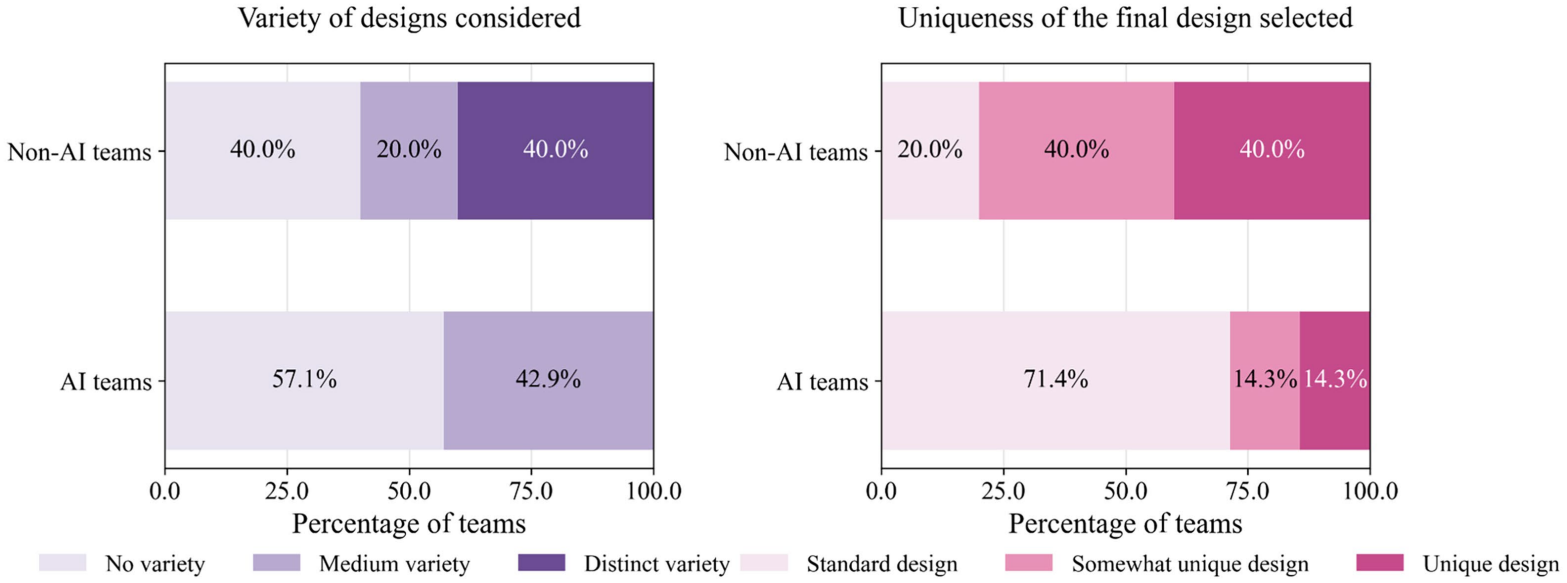
Variety of the designs considered, and uniqueness of the final design selected by AI-assisted and non-AI control teams.

Figure 2 presents the Variety and Uniqueness outcomes, revealing differences in creative processes. While mechanical variety of designs showed modest differences between conditions (40% vs. 29% achieving distinct principles with *U* = 11.5, *p* = 0.373, and *r* = −0.26), perceived design originality favored non-AI teams, with 40% rating their final designs as “very unique” compared to 14% (1 out of 7) of AI teams (*U* = 8.5, *p* = 0.168, and *r* = −0.40). These directional patterns align with the broader trend favoring human-driven creativity.

**Figure 3:**
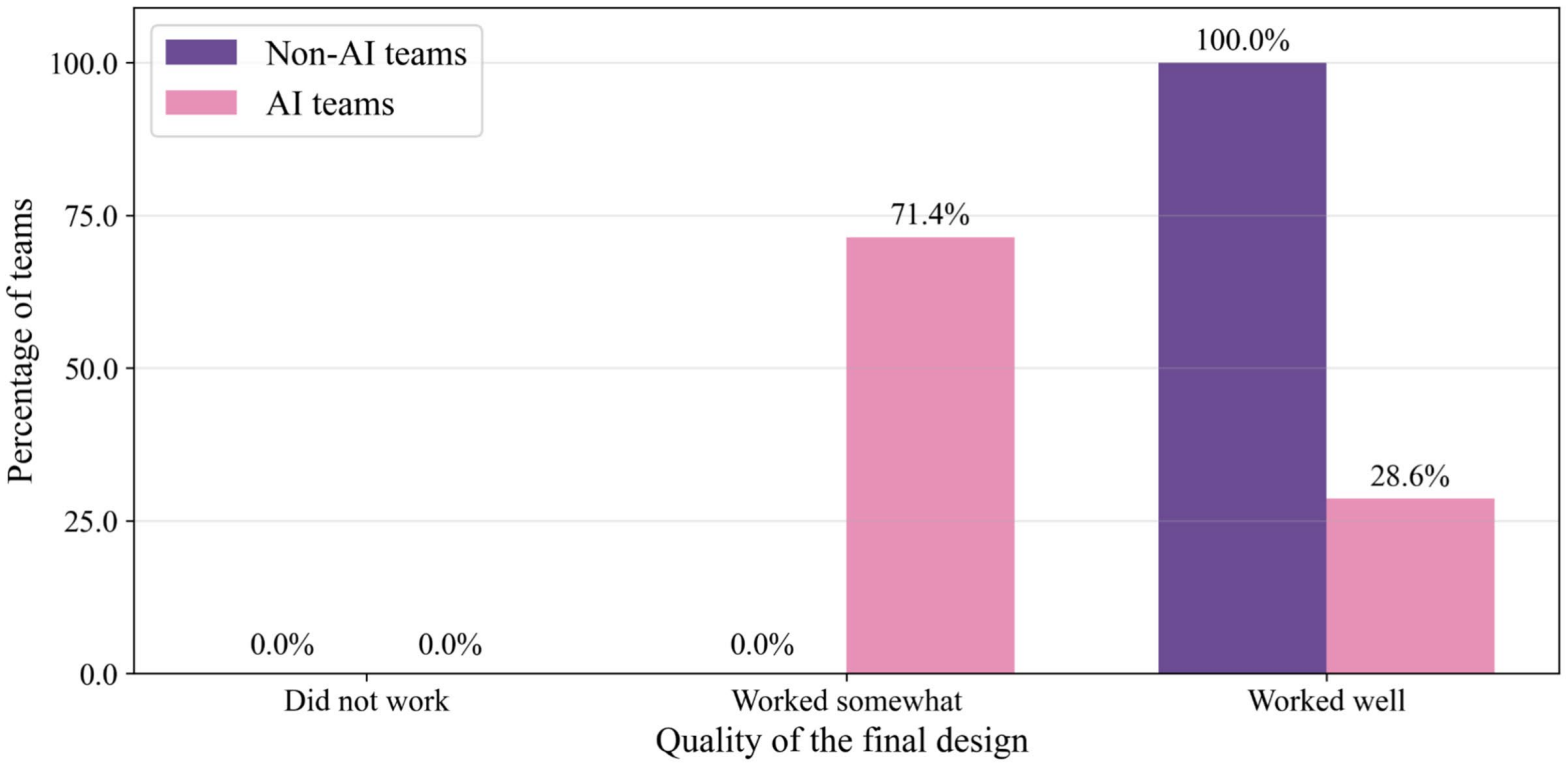
Quality of the final design assessed by AI-assisted and non-AI control teams.

The most pronounced difference emerged in functional performance, as depicted in Figure 3. All non-AI teams (100%) achieved catapults that “worked well,” while only 29% (2 out of 7) of AI-assisted teams reached this performance threshold (*U* = 5.0, *p* = 0.051, and *r* = −0.56). This substantial gap suggests that reliance on AI assistance may have compromised the iterative refinement process essential for achieving functional design solutions.

**Figure 4:**
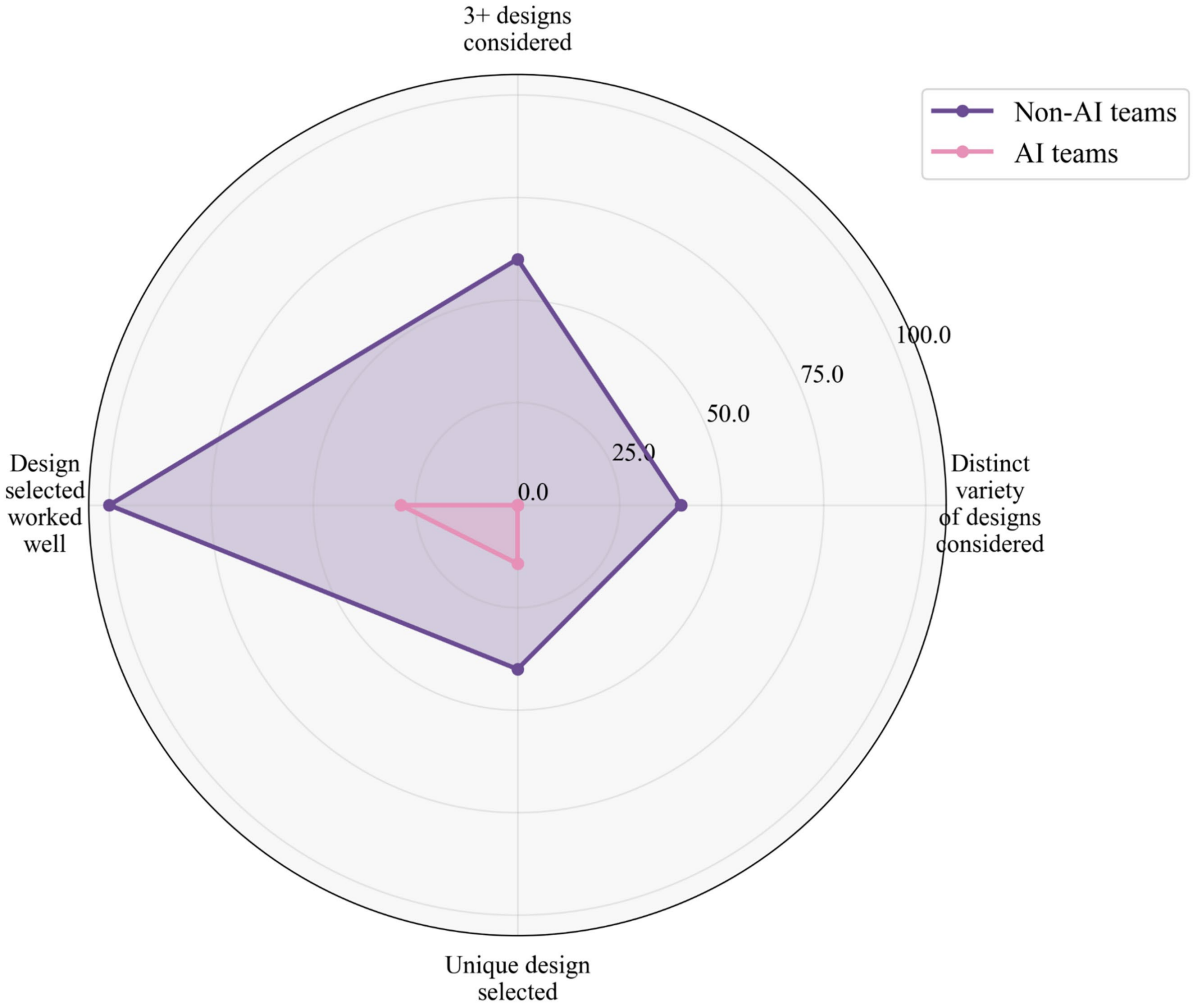
The four-dimensional framework for the team-level creativity assessments.

Figure 4 provides an integrated perspective through radar visualization, illustrating how non-AI teams consistently outperformed AI-assisted teams across the four creativity dimensions. The pattern reveals a particularly stark contrast in functional quality and design exploration, with more modest differences in variety and uniqueness measures. The observed patterns are consistent with premature convergence, in the sense that AI-assisted teams explored fewer alternatives and converged earlier on initial concepts.

### 3.2 Individual-level self-perceptions

Individual participants’ pre- to post-task changes in creative self-efficacy, divergent-thinking confidence, problem-solving efficacy, and innovative-thinking ratings were minimal in both conditions (Wilcoxon |*z*| < 1.2, *p* > 0.20 for all comparisons). This lack of change in self-perceived creative capability suggests several critical educational implications. First, the brief AI-assisted intervention did not enhance participants’ confidence in their creative abilities, despite the availability of AI support tools. Conversely, participants in AI-assisted teams did not experience diminished self-efficacy despite their objectively lower performance on team creativity measures. This disconnect between actual performance (where non-AI teams significantly outperformed AI teams) and self-perceived capability raises questions about participants’ awareness of AI’s impact on their creative processes.

The stability of individual creative self-perceptions across conditions suggests that participants may not have fully recognized how AI assistance affected their collaborative creative processes. This finding has implications for educational practice, suggesting that students may require explicit instruction on the potential benefits and limitations of AI tools in creative contexts, rather than assuming they will naturally develop appropriate usage strategies through experience alone.

### 3.3 Qualitative observations

Workshop observations revealed behavioral differences that explain the quantitative results. AI-assisted teams spent substantial time formulating prompts, interpreting ChatGPT responses, and adapting suggested concepts to fit available materials. This process consumed time that could otherwise be used for physical testing and design refinement. Non-AI teams proceeded directly to building prototypes, allowing for more iterative testing and improvement cycles.

The time-allocation patterns suggest that AI interaction created additional cognitive demands that competed with hands-on problem-solving. While AI-assisted teams received conceptual guidance, they struggled to translate abstract suggestions into functional designs within the time constraints. Non-AI teams, working without external input, focused their efforts on direct experimentation with materials and mechanisms, resulting in more effective final products.

## 4 Future studies and limitations

This study employed a quasi-experimental design in which participants self-organized into teams before being randomly assigned to AI-assisted and non-AI conditions. The condition assignment was randomized at the team level, and baseline equivalence was examined. We acknowledge the absence of full individual-level randomization introduces the possibility of self-selection effects and unobserved confounding variables that may affect internal validity. Accordingly, the findings should not be interpreted as establishing a causal effect of AI assistance on creativity outcomes. Rather, they indicate systematic associations and comparative patterns between AI-assisted and human-only teams within the specific constraints of a short, challenge-based workshop setting. Future research using fully randomized designs, crossover experiments, or longitudinal approaches would be required to support stronger causal inferences.

A further methodological limitation concerns the reliance on self-reported measures of creativity. These instruments, which capture participants’ perceived creative capability and self-perception data, are susceptible to bias, including overconfidence or miscalibration, particularly in AI-assisted contexts where external support may mask performance deficits. In this context, future studies should triangulate self-assessments with expert or peer evaluations. In addition, several key explanatory constructs discussed in this study, such as premature convergence and cognitive offloading, are introduced as theoretically grounded interpretive mechanisms. It is important to operationalize these inferred mechanisms more directly by incorporating process-level methods, such as think-aloud protocols, systematic process tracing of design decisions, or fine-grained interaction logs capturing human–AI collaboration dynamics over time. Such approaches would allow closer examination of how AI tools shape ideation trajectories, iteration cycles, and decision-making processes during creative problem-solving.

Finally, the study captures human–AI collaboration at a specific point in the (very) rapid evolution of generative AI technologies and within a constrained time frame. Replication across different task durations, disciplinary contexts, and with newer generations of AI systems would help assess the robustness and generalizability of the observed patterns.

## 5 Discussion and conclusions

Before interpreting these findings, it is important to clarify the scope of inference permitted by the study design. Although teams were randomly assigned to AI-assisted and non-AI conditions, team formation occurred through self-organization prior to condition assignment. As a result, the quasi-experimental design does not support strong causal claims regarding the effects of AI assistance on creativity outcomes. The patterns discussed below should therefore be interpreted as comparative and associational, reflecting differences observed between AI-assisted and human-only teams under the specific constraints of a short, challenge-based workshop, rather than as evidence of direct causal effects.

Our findings suggest that AI assistance in team-based design tasks is associated with patterns consistent with premature convergence, leading to fewer explored alternatives and lower functional quality compared to human-only teams. While AI tools offered structured suggestions, they often constrained divergent exploration, limiting opportunities for iteration and refinement. This pattern is consistent with prior findings in AI-enhanced software development education, where large language models were found to accelerate early-stage ideation and task execution, while raising concerns about customization, transparency, and depth of engagement in later stages of development (Israilidis et al., 2024).

Cutting-edge research argues that current AI systems operate as fragmented utilities rather than process-aware collaborators (Wang and Lu, 2025). Their proposed layered framework emphasizes that effective human–AI collaboration requires explicit process representation and adaptability over time. Our results illustrate a pattern in which, when such support is absent, what happens when such support is absent: students rely on AI for isolated outputs but lose the iterative, hands-on engagement that supports deeper problem-solving.

At the same time, the Memory Paradox (Oakley et al., 2025) highlights how excessive reliance on external aids can erode internal cognitive development, weakening schema formation and procedural fluency. In our study, participants in AI-assisted teams maintained confidence in their creative abilities despite producing weaker outcomes, reflecting the risk that AI offloading may mask shallow engagement. Without building strong internal “creative schemata” through practice and reflection, learners will ultimately fail to develop transferable problem-solving skills. This perception–performance gap also highlights a methodological limitation or relying solely on self-reported measures, which may now capture actual creative performance.

The findings can be interpreted through a broader human-centered theoretical lens on creativity. Lockhart (2025) argues that creativity is not reducible to the generation of novel outputs but is fundamentally grounded in lived experience, embodied understanding, and moral agency. From this perspective, creative acts emerge through situated engagement with materials, constraints, and failure, rather than through abstract recombination alone.

Nevertheless, taken together, these observations point to a central tension: AI may assist in accelerating access to ideas, but without structured collaboration and deliberate memory-building, it risks narrowing exploration and undermining genuine learning. The challenge is not only to integrate AI responsibly but also to design environments that preserve human agency, iterative refinement, and the development of durable creative capacities.

## Data Availability

The raw data supporting the conclusions of this article will be made available by the authors without undue reservation.
