# Supplementary material for: AI-assisted design synthesis and human creativity in engineering education
Source: Front Artif Intell. 2026 Jan 20;9:1714523. doi: 10.3389/frai.2026.1714523 (PMC12864478; doi:10.3389/frai.2026.1714523)
Supplement: Supplementary file 1 [file Data_Sheet_1.docx]

# Appendix A: Photographic Documentation of Workshop Set-up and Prototype Construction Environment


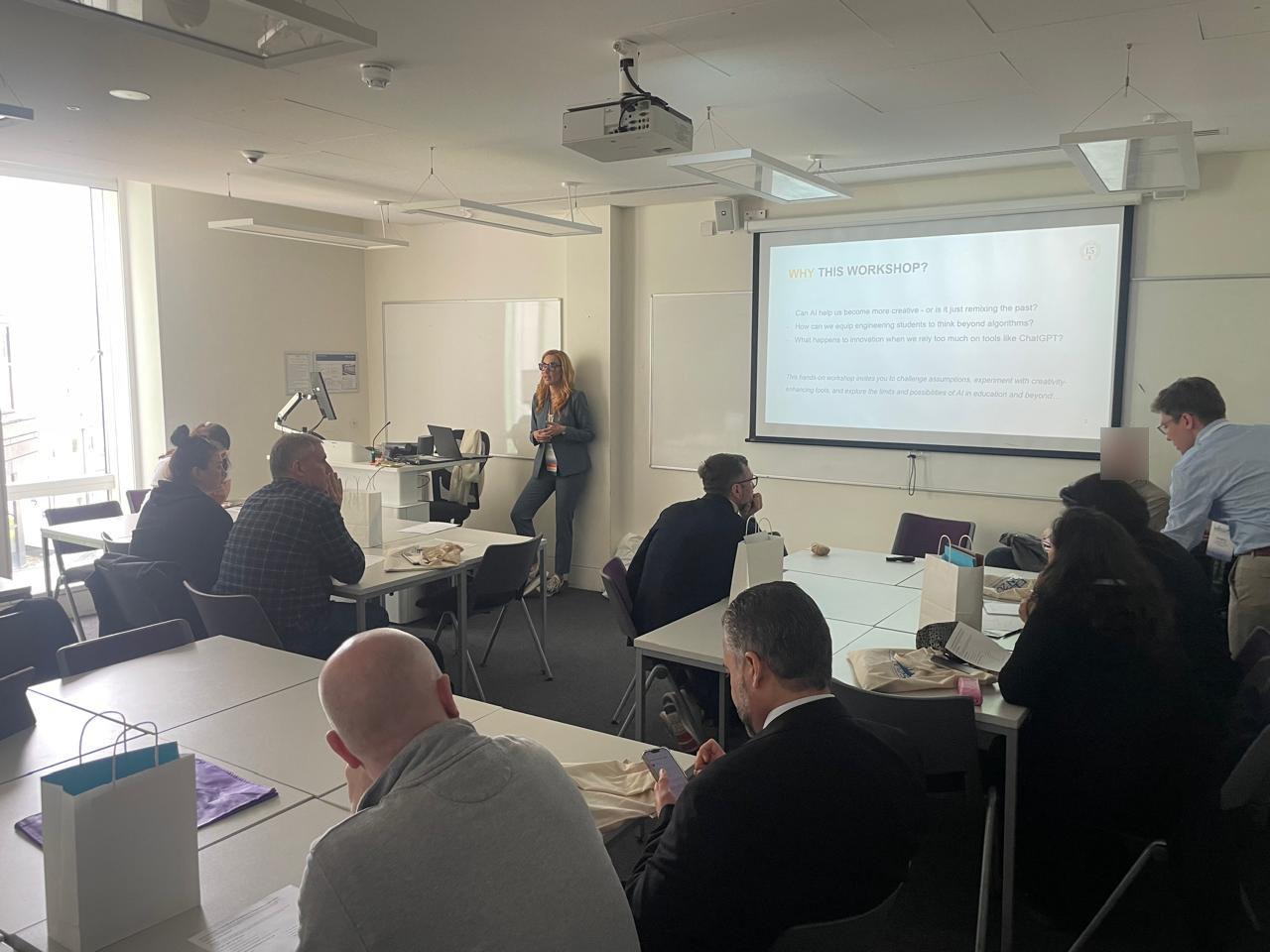

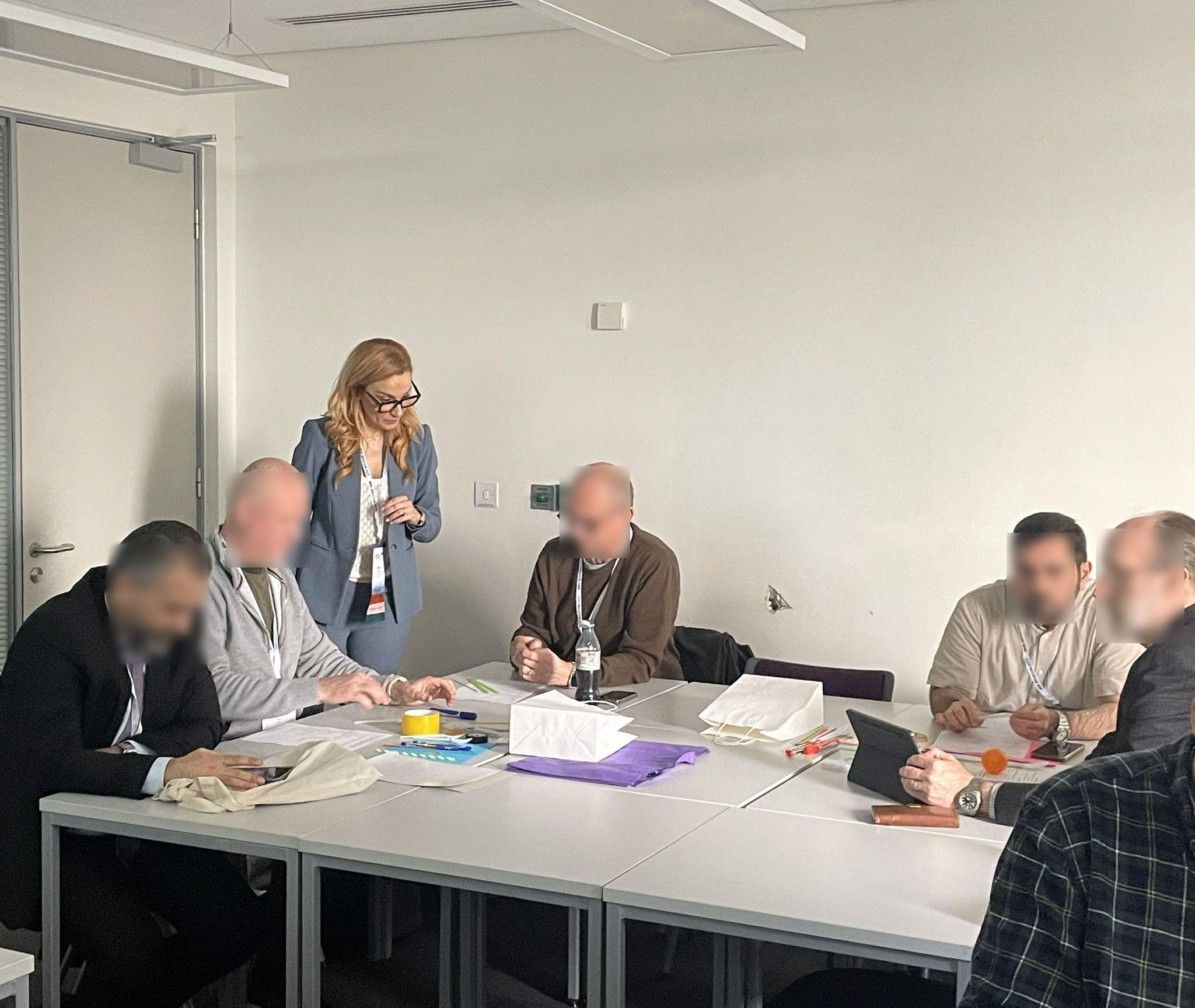

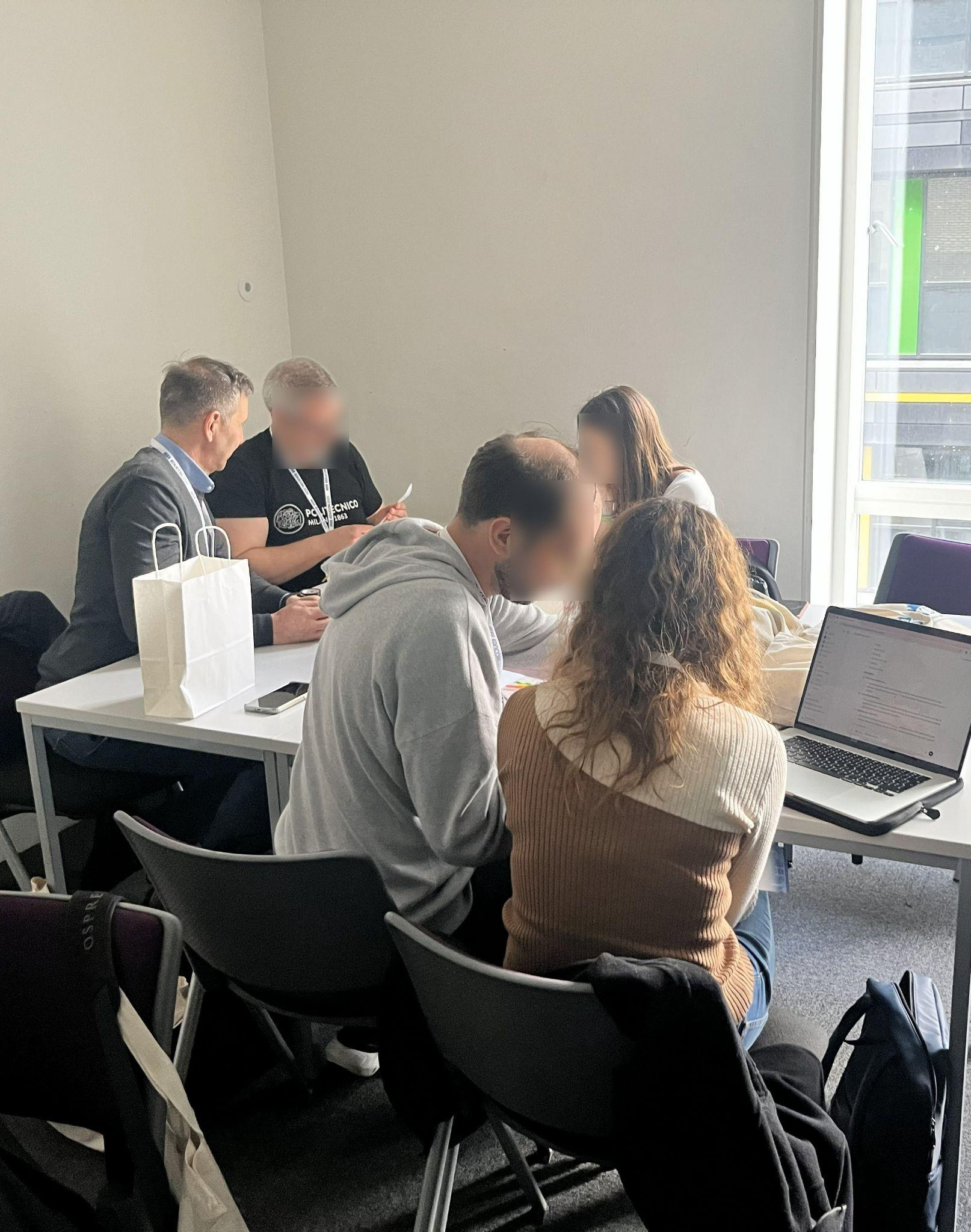

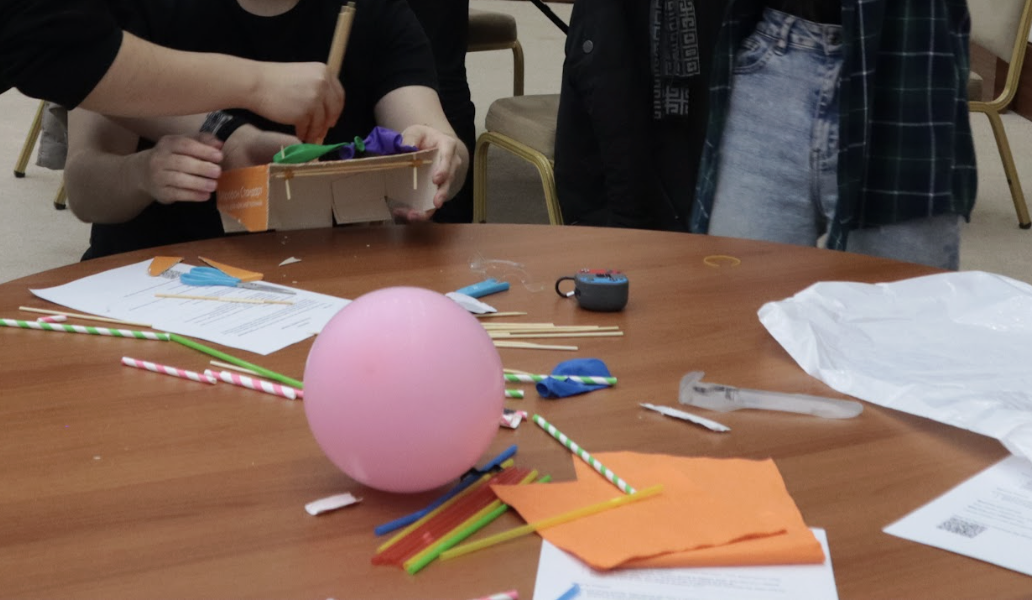

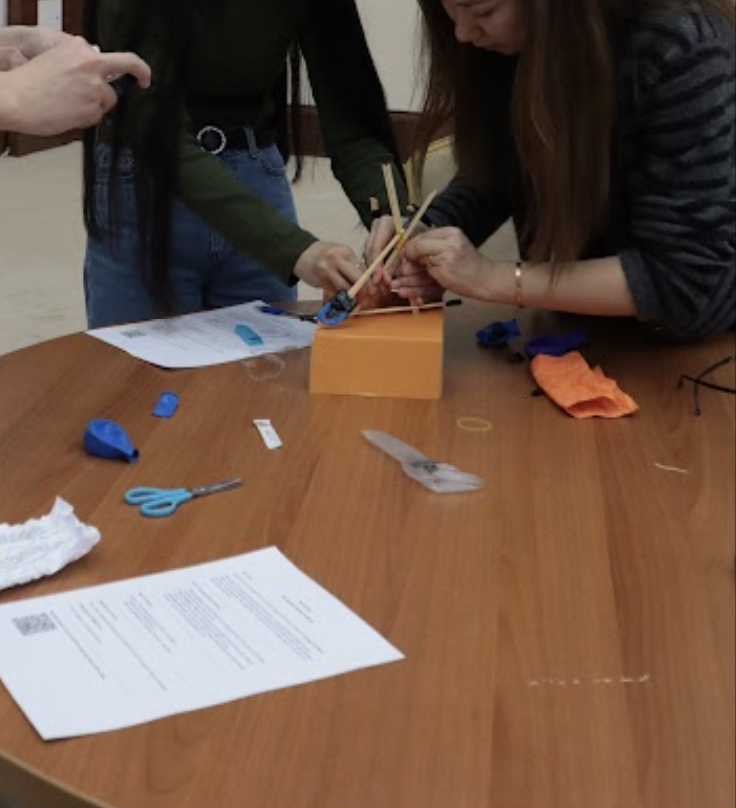

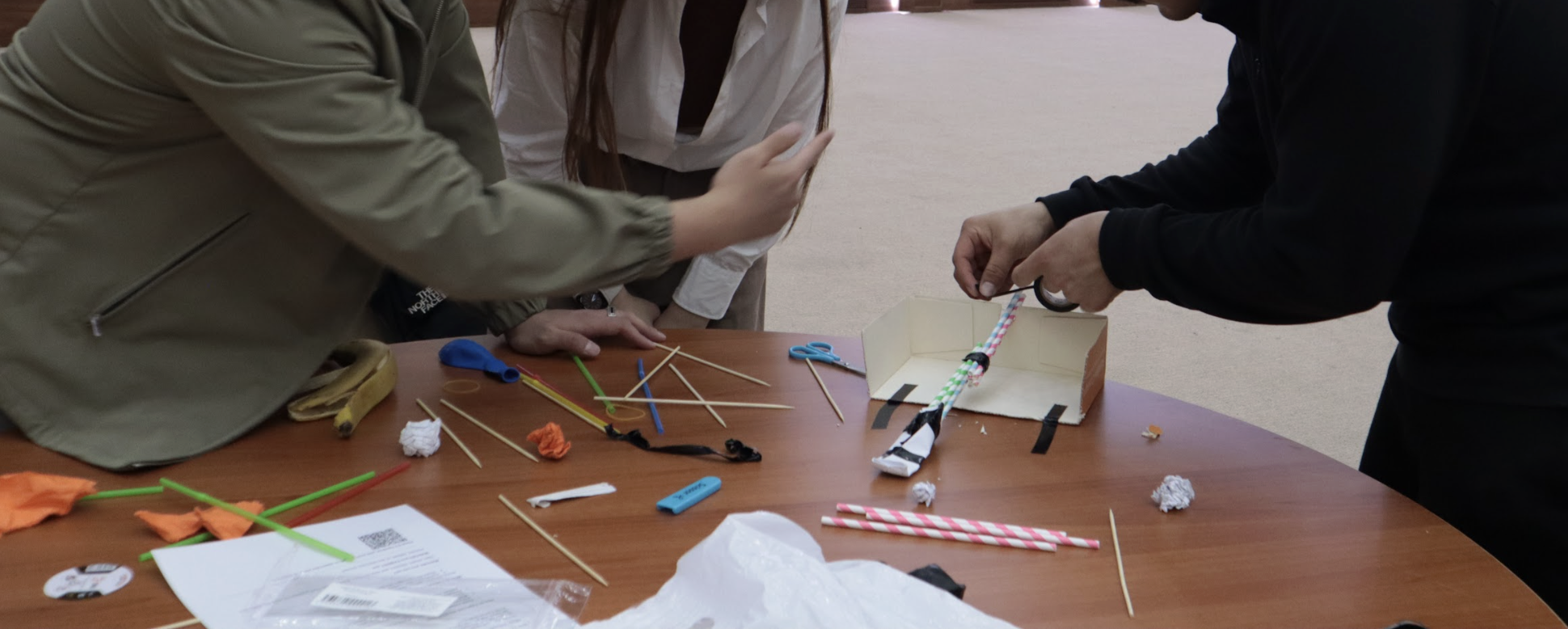

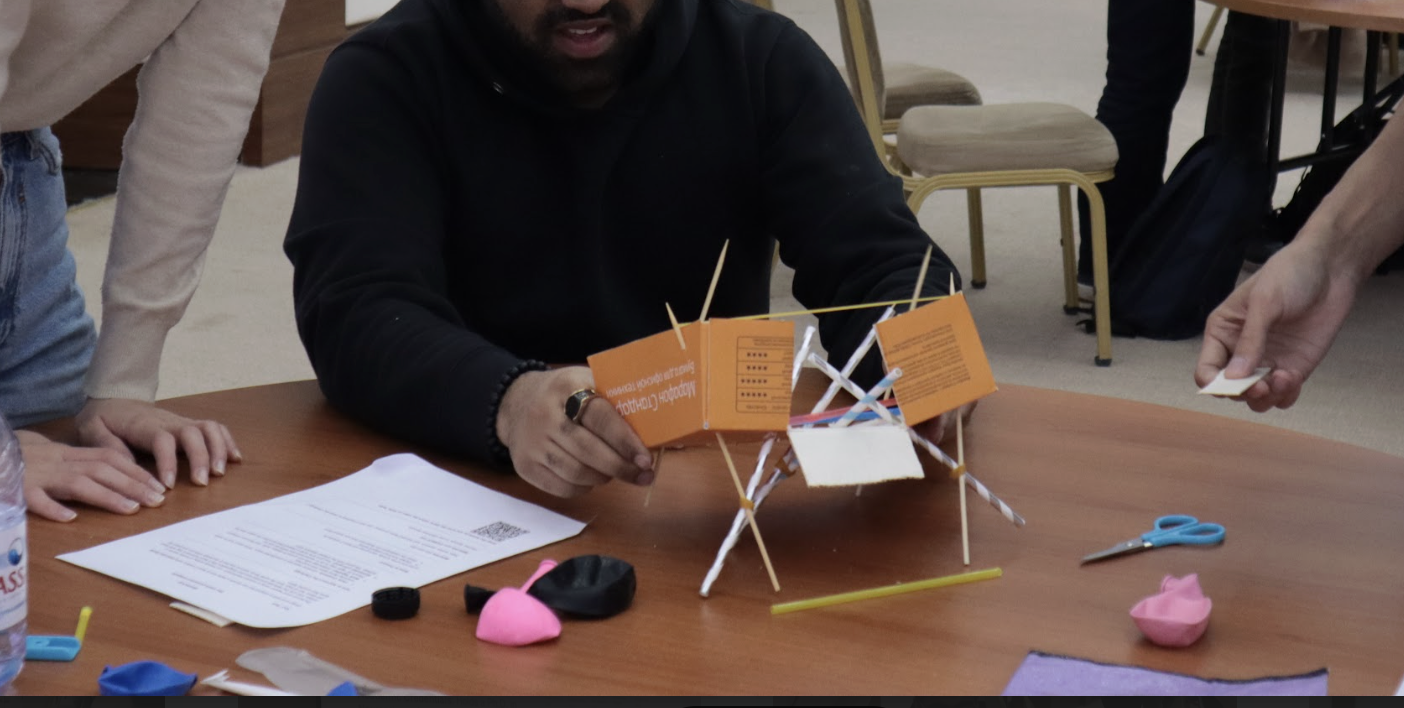


Figure A1. Workshop environment during the Catapult Challenge. Images illustrate the construction process, materials, and testing setup used in the study. Individual participants are shown only incidentally, and no identifiable data were used in the analysis.

**Appendix B: Survey Questions**

Creativity Pre-Workshop survey

Section 1. Demographic data

1. Please indicate your age:

- Under 18
- 18–24
- 25–34
- 35–44
- 45–54
- 55–64
- 65+

2. Please indicate your gender:

- Male
- Female
- Non-binary / third gender
- Prefer not to say

3. What is your field of study or professional background?

- Engineering
- Science
- Arts/Humanities
- Business
- Environmental and Natural Resource Management
- Sustainable Development and Policy
- Geography and Science
- Other:

Section 2. Self-reflection questions

Creative self-efficacy*:

1. I am confident in my ability to generate original ideas when solving complex problems.
   1 = Strongly disagree; 2 = Disagree; 3 = Neither disagree nor agree; 4 = Agree; 5 = Strongly agree
2. I believe that my creative thinking skills help me excel academically or professionally.
   1 = Strongly disagree; 2 = Disagree; 3 = Neither disagree nor agree; 4 = Agree; 5 = Strongly agree
3. I am confident in expressing my creative ideas to others, even in professional settings.
   1 = Strongly disagree; 2 = Disagree; 3 = Neither disagree nor agree; 4 = Agree; 5 = Strongly agree

Divergent thinking** capabilities:

1. I can easily generate multiple ideas when faced with a problem.
   1 = Strongly disagree; 2 = Disagree; 3 = Neither disagree nor agree; 4 = Agree; 5 = Strongly agree
2. I tend to come up with different types of solutions (e.g., practical, artistic, technical) when brainstorming.
   1 = Strongly disagree; 2 = Disagree; 3 = Neither disagree nor agree; 4 = Agree; 5 = Strongly agree
3. I enjoy approaching problems with a wide range of ideas rather than sticking to one solution.
   1 = Strongly disagree; 2 = Disagree; 3 = Neither disagree nor agree; 4 = Agree; 5 = Strongly agree

Problem-solving abilities:

1. When solving problems, I strive to find solutions that are both creative and practical.
   1 = Strongly disagree; 2 = Disagree; 3 = Neither disagree nor agree; 4 = Agree; 5 = Strongly agree
2. I feel confident in finding new approaches when conventional solutions are not effective.
   1 = Strongly disagree; 2 = Disagree; 3 = Neither disagree nor agree; 4 = Agree; 5 = Strongly agree
3. I am resourceful in using limited resources or time to solve complex problems.
   1 = Strongly disagree; 2 = Disagree; 3 = Neither disagree nor agree; 4 = Agree; 5 = Strongly agree

Innovative thinking:

1. I am able to think of innovative solutions when given complex, real-world challenges.
   1 = Strongly disagree; 2 = Disagree; 3 = Neither disagree nor agree; 4 = Agree; 5 = Strongly agree
2. I feel capable of coming up with ideas that have the potential to make a significant impact.
   1 = Strongly disagree; 2 = Disagree; 3 = Neither disagree nor agree; 4 = Agree; 5 = Strongly agree
3. I am able to elaborate on and refine my creative ideas to ensure they are effective and impactful.
   1 = Strongly disagree; 2 = Disagree; 3 = Neither disagree nor agree; 4 = Agree; 5 = Strongly agree

*Self-efficacy is a person’s belief in their ability to succeed in completing tasks or achieving goals, influencing their persistence and motivation.
**Divergent thinking, often referred to as lateral thinking, is the process of generating multiple, unique ideas or solutions to a problem in an open-ended and creative manner.

Creativity Post-Workshop survey

Section 1. Self-reflection questions

Creative self-efficacy*:

1. I am confident in my ability to generate original ideas when solving complex problems.
   1 = Strongly disagree; 2 = Disagree; 3 = Neither disagree nor agree; 4 = Agree; 5 = Strongly agree
2. I believe that my creative thinking skills help me excel academically or professionally.
   1 = Strongly disagree; 2 = Disagree; 3 = Neither disagree nor agree; 4 = Agree; 5 = Strongly agree
3. I am confident in expressing my creative ideas to others, even in professional settings.
   1 = Strongly disagree; 2 = Disagree; 3 = Neither disagree nor agree; 4 = Agree; 5 = Strongly agree

Divergent thinking** capabilities:

1. I can easily generate multiple ideas when faced with a problem.
   1 = Strongly disagree; 2 = Disagree; 3 = Neither disagree nor agree; 4 = Agree; 5 = Strongly agree
2. I tend to come up with different types of solutions (e.g., practical, artistic, technical) when brainstorming.
   1 = Strongly disagree; 2 = Disagree; 3 = Neither disagree nor agree; 4 = Agree; 5 = Strongly agree
3. I enjoy approaching problems with a wide range of ideas rather than sticking to one solution.
   1 = Strongly disagree; 2 = Disagree; 3 = Neither disagree nor agree; 4 = Agree; 5 = Strongly agree

Problem-solving abilities:

1. When solving problems, I strive to find solutions that are both creative and practical.
   1 = Strongly disagree; 2 = Disagree; 3 = Neither disagree nor agree; 4 = Agree; 5 = Strongly agree
2. I feel confident in finding new approaches when conventional solutions are not effective.
   1 = Strongly disagree; 2 = Disagree; 3 = Neither disagree nor agree; 4 = Agree; 5 = Strongly agree
3. I am resourceful in using limited resources or time to solve complex problems.
   1 = Strongly disagree; 2 = Disagree; 3 = Neither disagree nor agree; 4 = Agree; 5 = Strongly agree

Innovative thinking:

1. I am able to think of innovative solutions when given complex, real-world challenges.
   1 = Strongly disagree; 2 = Disagree; 3 = Neither disagree nor agree; 4 = Agree; 5 = Strongly agree
2. I feel capable of coming up with ideas that have the potential to make a significant impact.
   1 = Strongly disagree; 2 = Disagree; 3 = Neither disagree nor agree; 4 = Agree; 5 = Strongly agree
3. I am able to elaborate on and refine my creative ideas to ensure they are effective and impactful.
   1 = Strongly disagree; 2 = Disagree; 3 = Neither disagree nor agree; 4 = Agree; 5 = Strongly agree

*Self-efficacy is a person’s belief in their ability to succeed in completing tasks or achieving goals, influencing their persistence and motivation.
**Divergent thinking, often referred to as lateral thinking, is the process of generating multiple, unique ideas or solutions to a problem in an open-ended and creative manner.

Section 2.1. Main assessment (AI team)

1. How many different catapult designs did your team consider?

- 1 design
- 2 designs
- 3 designs
- 4 designs
- 5 or more designs

2. Did your team explore different mechanical approaches?

- No - all designs used the same mechanical principle
- Somewhat - designs used variations of the same principle
- Yes - designs employed distinctly different principles

3. How unique was your final catapult compared to others?

- Standard design
- Somewhat unique
- Very unique

4. How well did your catapult work?

- Did not work
- Worked somewhat
- Worked well

5. How did AI tools affect your team's overall performance?

- Significantly hindered
- Slightly hindered
- No impact
- Somewhat improved
- Greatly improved

6. How helpful was AI for generating new ideas (divergent thinking)?

- Did not use AI for ideas
- Not helpful
- Somewhat helpful
- Very helpful

7. How helpful was AI for refining your design (convergent thinking)?

- Did not use AI for refinement
- Not helpful
- Somewhat helpful
- Very helpful

Section 2.2. Main assessment (non-AI team)

1. How many different catapult designs did your team consider?

- 1 design
- 2 designs
- 3 designs
- 4 designs
- 5 or more designs

2. Did your team explore different mechanical approaches?

- No - all designs used the same mechanical principle
- Somewhat - designs used variations of the same principle
- Yes - designs employed distinctly different principles

3. How unique was your final catapult compared to others?

- Standard design
- Somewhat unique
- Very unique

4. How well did your catapult work?

- Did not work
- Worked somewhat
- Worked well
